# Small‐Molecule‐Induced and Cooperative Enzyme Assembly on a 14‐3‐3 Scaffold

**DOI:** 10.1002/cbic.201600631

**Published:** 2016-12-27

**Authors:** Anniek den Hamer, Lenne J. M. Lemmens, Minke A. D. Nijenhuis, Christian Ottmann, Maarten Merkx, Tom F. A. de Greef, Luc Brunsveld

**Affiliations:** ^1^ Laboratory of Chemical BiologyDepartment of Biomedical Engineering andInstitute of Complex Molecular SystemsEindhoven University of TechnologyDen Dolech 25612AZ EindhovenNetherlands

**Keywords:** 14-3-3 proteins, combinatorial inhibition, cooperative effects, dimerization, protein engineering, protein scaffolds

## Abstract

Scaffold proteins regulate cell signalling by promoting the proximity of putative interaction partners. Although they are frequently applied in cellular settings, fundamental understanding of them in terms of, amongst other factors, quantitative parameters has been lagging behind. Here we present a scaffold protein platform that is based on the native 14‐3‐3 dimeric protein and is controllable through the action of a small‐molecule compound, thus permitting study in an in vitro setting and mathematical description. Robust small‐molecule regulation of caspase‐9 activity through induced dimerisation on the 14‐3‐3 scaffold was demonstrated. The individual parameters of this system were precisely determined and used to develop a mathematical model of the scaffolding concept. This model was used to elucidate the strong cooperativity of the enzyme activation mediated by the 14‐3‐3 scaffold. This work provides an entry point for the long‐needed quantitative insights into scaffold protein functioning and paves the way for the optimal use of reengineered 14‐3‐3 proteins as chemically inducible scaffolds in synthetic systems.

## Introduction

Scaffold proteins act as signalling hubs in eukaryotic signalling pathways by co‐localising other proteins. Cellular studies have revealed that scaffold proteins are able to regulate the speed, amplitude, sensitivity and specificity of signal transduction in the intracellular environment.^1^ Synthetically engineered molecular scaffolds are important tools for bottom‐up synthetic biology, as they allow engineering of new pathway behaviour by mediating pathway regulation and feedback.^2^ The concept of small‐molecule‐induced control over protein–protein interactions potentially permits remote ON/OFF switching,^3^ thereby enlarging the synthetic toolbox. The possibility of rewiring or controlling scaffolds shows promising results in customised regulation of signalling pathways.^4^, ^5^ Nevertheless, the number of tractable protein‐based scaffold systems is highly limited,^1^, ^6^, ^7^ and well‐characterised scaffold proteins under the control of small‐molecule regulation remain effectively unknown.

Cell‐free synthetic biology similarly requires fully controllable scaffold systems.^8^, ^9^, ^10^ Importantly, an exact understanding and application of the scaffolding concept requires quantitative models that provide detailed insight in the physical/chemical parameters that determine scaffolding activity, such as concentration dependence and cooperativity. Existing models are scarce^11^, ^12^ and typically formulated on the basis of cellular data, not providing exact and quantitative physical/chemical data. Also, these models have not yet considered small‐molecule stabilisation systems. As such, there is a strong need for well‐defined protein scaffolds that are easily accessible for in vitro studies, controllable through diverse chemical input, including small‐molecule compounds and mutations, and which can be described by quantitative mathematical models.

The 14‐3‐3 proteins are a family of natural dimeric proteins that bind to specific peptide sequences featuring a phosphorylated serine/threonine residue.^13^ One of their natural functions is to facilitate protein–protein interactions by scaffolding;^14^, ^15^, ^16^ moreover, the binding of 14‐3‐3 proteins to some of their interaction partners has been shown to be amenable to small‐molecule stabilisation.^17^ The versatility of the 14‐3‐3 protein dimer gives it potential as an engineered synthetic scaffold under the control of small‐molecule input; a first example of which we recently demonstrated in its potential to control intracellular NF‐κB localisation.^18^ The 14‐3‐3 proteins thus constitute an ideal platform on which to assess quantitatively, for the first time, the parameters playing a role in scaffold functioning. The potential for engineering of designed scaffold systems based on 14‐3‐3 provides an entry point for their study in controlled in vitro settings, which in turn allows the formulation of mathematical models based on the determined data.

**Scheme 1:**
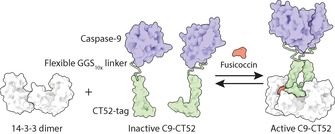
Schematic representation of the chemically induced scaffold system consisting of a 14‐3‐3 dimer and a recombinant fusion protein of caspase‐9 fused to a CT52 domain (C9‐CT52). Upon addition of the small‐molecule stabiliser fusicoccin (FC), two C9‐CT52 units assemble on the scaffold, and this leads to caspase‐9 activation through dimerisation and a concomitant increase in enzymatic activity.

In a bottom‐up approach we therefore fundamentally explored the characteristics and potential of 14‐3‐3 proteins as scaffold proteins. A 14‐3‐3 protein was engineered as a dimerisation scaffold for enzyme assembly and activation. The mode of action and potential of this small‐molecule‐controlled scaffolding concept was revealed and characterised through a combination of in vitro studies and mathematical modelling. We chose the widely studied caspase‐9 (C9) enzyme as proof‐of‐principle for the readout of our model system, because of its large increase in activity upon dimerisation^19^, ^20^, ^21^ and the availability of fluorogenic substrates,^22^ allowing for a quantitative analysis of the dimeric scaffolding of monomeric C9 on 14‐3‐3 scaffolds. Monomeric C9 constructs were fused to the C‐terminal part (CT52) of the 14‐3‐3 interaction partner plant plasma membrane H^+^‐ATPase (PMA2). Importantly, the native threonine phosphorylation site in CT52 was replaced by a phosphomimetic aspartic acid, thus rendering the interaction between CT52 and engineered plant 14‐3‐3^15^ solely and critically dependent on the presence of the small‐molecule compound fusicoccin (FC).^23^ To allow for optimal proximity of the C9 domains on the 14‐3‐3 scaffold, a flexible GGS linker of ten repeats was introduced between the C terminus of the C9 domain and the N terminus of CT52 (Scheme 1).

## Results and Discussion

### Activity assay with synthetic substrate Ac‐LEHD‐AFC

**Figure 1:**
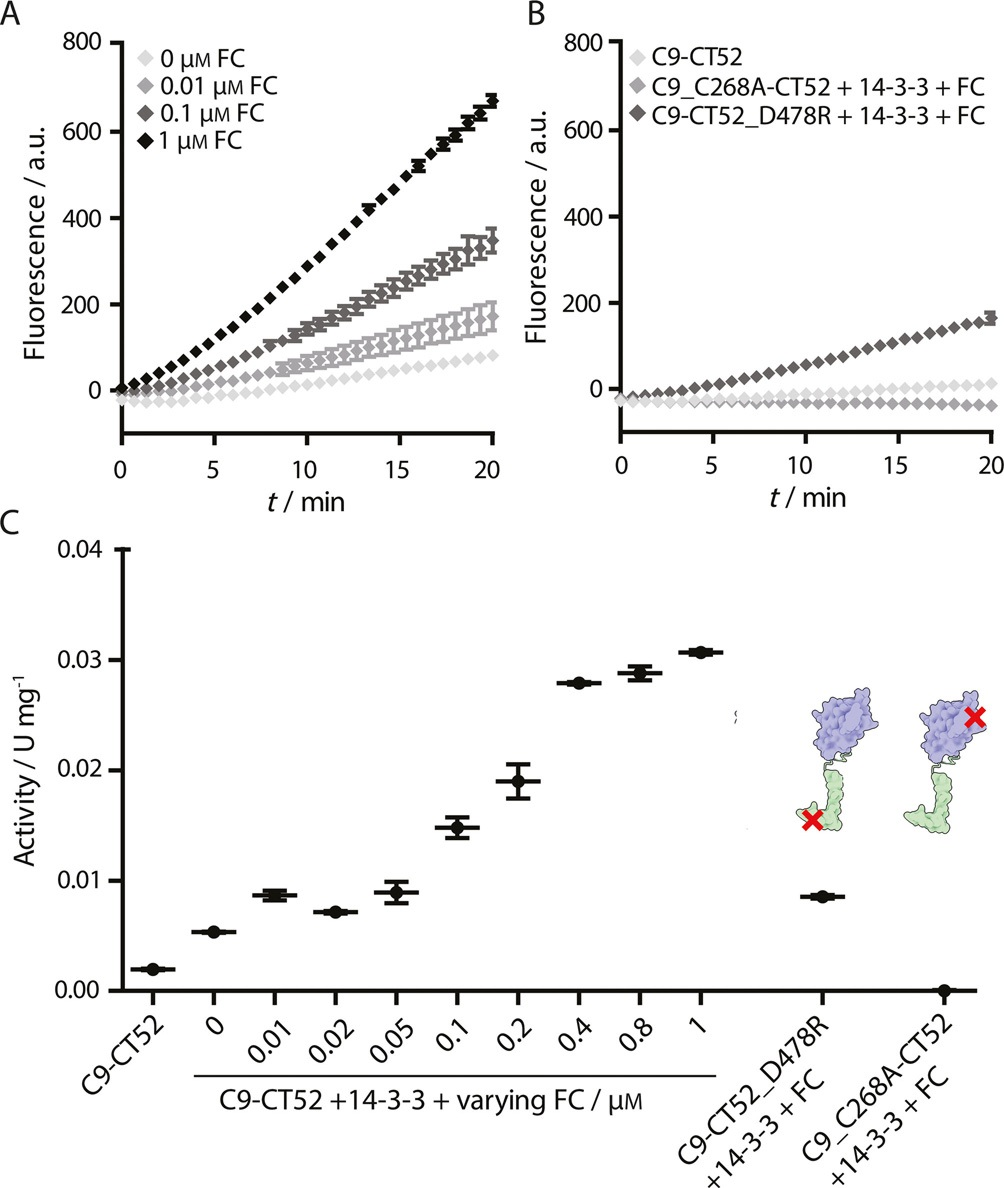
14‐3‐3‐scaffolded and small‐molecule‐induced caspase‐9 dimerisation probed by cleavage of a fluorogenic substrate. A) Fluorescent traces corresponding to cleavage of synthetic substrate Ac‐LEHD‐AFC (200 μm) in the presence of C9‐CT52 (0.1 μm), 14‐3‐3 (1 μm) and varying concentrations of FC (0, 0.01, 0.1 and 1 μm). B) Fluorescent traces corresponding to the following experiments: i) 0.1 μm C9‐CT52, ii) 0.1 μm C9_C268R‐CT52, 1 μm 14‐3‐3, and 1 μm FC, and iii) 0.1 μm C9‐CT52_D478R, 1 μm 14‐3‐3, and 1 μm FC. Ac‐LEHD‐AFC concentration is 200 μm in all experiments. C) Activity of caspase‐9 proteins (U mg^−1^) determined from the traces in (A) and (B). All experiments were conducted at 37 °C in assay buffer (20 mm Na_2_HPO_4_, 150 mm NaCl, 1 mm EDTA, 2 mm TCEP, pH 7.0). Each error bar represents the standard deviation in (A) and (B) and the standard error of the mean in (C) based on three independent measurements.

The 14‐3‐3 scaffold protein and the C9‐CT52 fusion protein were expressed in *E. coli* cells and purified by Ni‐affinity chromatography (Supporting Information). Dimerisation and subsequent activation of C9‐CT52 was examined by use of the synthetic fluorogenic substrate Ac‐LEHD‐AFC (*N*‐acetyl‐Leu‐Glu‐His‐Asp‐7‐amino‐4‐trifluoromethyl coumarin; Figure 1). The activity (U mg^−1^) of the recombinant C9 was calculated from the initial rate of the fluorescent traces by use of a calibration curve (Figure 1 C and Figure S4 in the Supporting Information).

A 16‐fold increase in C9‐CT52 activity was observed upon addition of 14‐3‐3 scaffold and small‐molecule compound FC. Addition of 14‐3‐3 alone to C9‐CT52 led only to a 2.7‐fold increase in activity (Figure 1 C). Increasing concentrations of FC (0.01–1 μm) in the presence of 0.1 μm C9‐CT52 and 1 μm 14‐3‐3 led to controllable enhancement in enzyme activity (Figure 1 A). FC thus functions as an input in controlling the 14‐3‐3 scaffold function.

Mutated C9‐CT52 variants were evaluated as reference constructs. Inactivated caspase C9_C268A‐CT52 featured no activity, even in the presence of 1 μm FC and 14‐3‐3, thus clearly revealing that substrate cleavage is solely due to active C9. Mutation of the phosphate‐mimicking D478 in the CT52 domain to an arginine (C9‐CT52_D478R), efficiently suppressed the 14‐3‐3‐mediated C9 activation even in the presence of 1 μm FC, showing activity levels similar to those of C9‐CT52/14‐3‐3 in the absence of FC. The phosphate‐mimicking aspartic acid D478 is essential for high‐affinity binding into the 14‐3‐3 binding groove and resulting dimer formation by C9‐CT52. Tuning the 14‐3‐3 binding affinity of the C9 construct thus allows the strength of the functional output to be controlled.

### Activity assay with natural substrate caspase‐3

**Figure 2:**
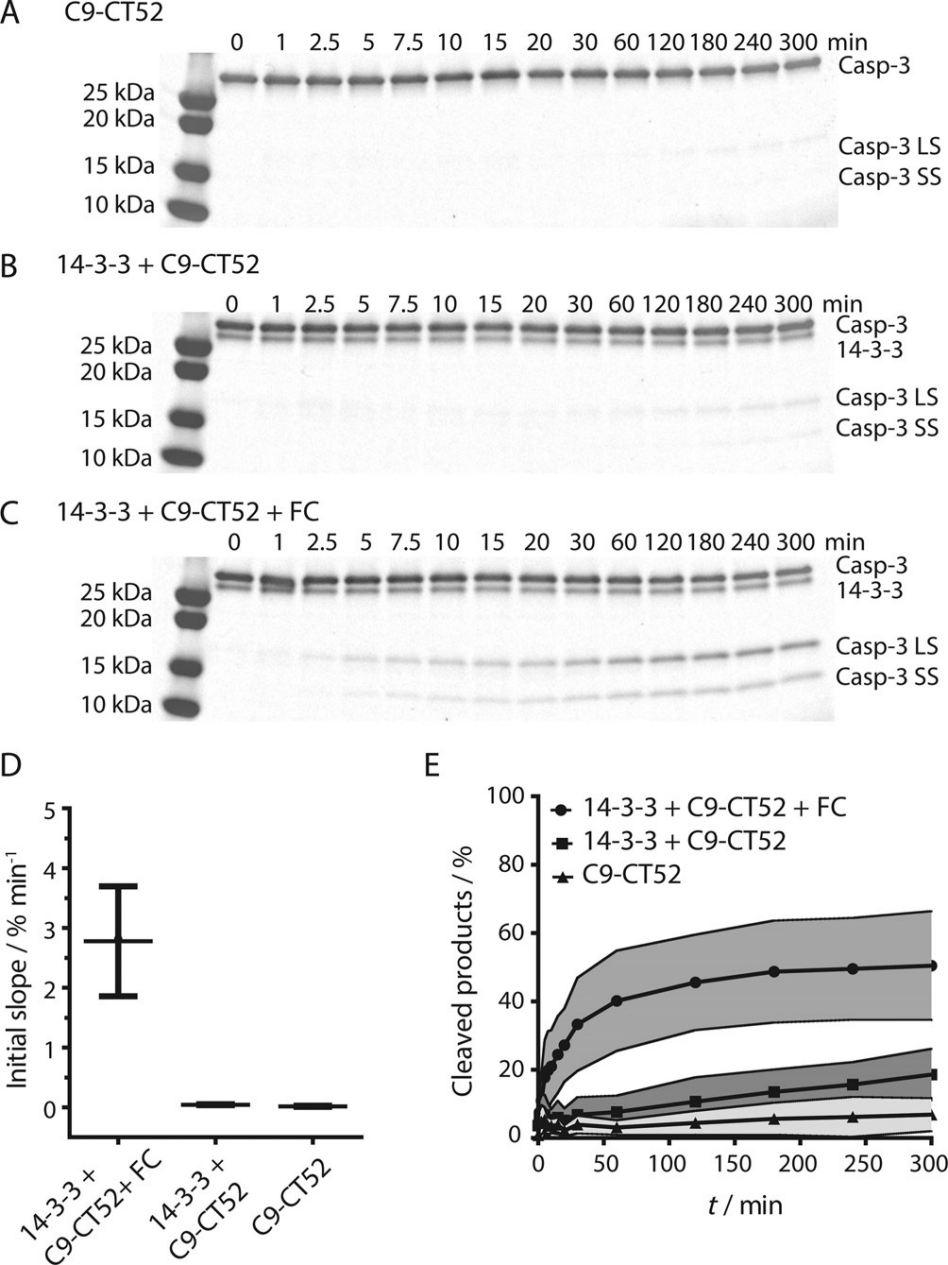
Small‐molecule‐induced caspase‐9‐mediated cleavage of the natural substrate caspase‐3_C158A. A)–C) SDS‐PAGE gels showing cleavage of caspase‐3_C158A (4 μm) into its large subunit (Casp‐3 LS) and small subunit (Casp‐3 SS) by A) 0.1 μm C9‐CT52, B) 0.1 μm C9‐CT52 and 1 μm 14‐3‐3, and C) 0.1 μm C9‐CT52, 1 μm 14‐3‐3 and 1 μm FC. D) Initial slopes of the activity assays in (A)–(C). Each error bar represents the standard error of the mean (*n*=3). E) Quantification of caspase‐3_C158A (*n*=3) cleavage showing the percentages of cleaved products (Casp‐3 LS and Casp‐3 SS) over time in the presence of FC (grey) and in the absence of FC (dark grey) and the background activity of C9‐CT52 (light grey). Each error bar represents the standard deviation (*n*=3).

During apoptosis, caspase‐9 cleaves its downstream substrate caspase‐3, leading to caspase‐3 activation and subsequent cleavage of substrates further downstream, eventually leading to apoptosis.^24^ To evaluate the 14‐3‐3 scaffolding concept on the natural signalling substrate, caspase‐3 cleavage assays were performed. For this, a caspase‐3_C158A mutant that does not display auto‐cleavage capacity was generated.^25^ SDS‐PAGE analysis was used to gauge the effect of 14‐3‐3 scaffolding on C9‐CT52 enzymatic activity (Figure 2 A–C). The analysis of the initial enzymatic reaction rates reveals that the combined presence of both FC and 14‐3‐3 greatly enhances the C9‐CT52 activity (Figure 2 D) as indicated by the 60‐fold enhancement in activity in relation to the same reaction in the absence of 14‐3‐3 and/or FC. The highly efficient caspase‐9 activity in the presence of both FC and 14‐3‐3 was already resulting in a maximum of about 40 % caspase‐3 cleavage after 1 h. The cleavage of caspase‐3 did not reach full conversion, most probably because of product inhibition.^26^ Even after 6 h, the background activity of C9‐CT52 alone, resulting from the intrinsic dimerisation of caspase‐9,^19^ led to only 7 % cleaved caspase‐3_C158A (Figure 2 E). The addition of 14‐3‐3 to C9‐CT52, in the absence of FC, slightly enhanced activity, resulting in 18 % of the natural substrate being cleaved after six hours.

### Cooperativity in 14‐3‐3 scaffolding

**Figure 3:**
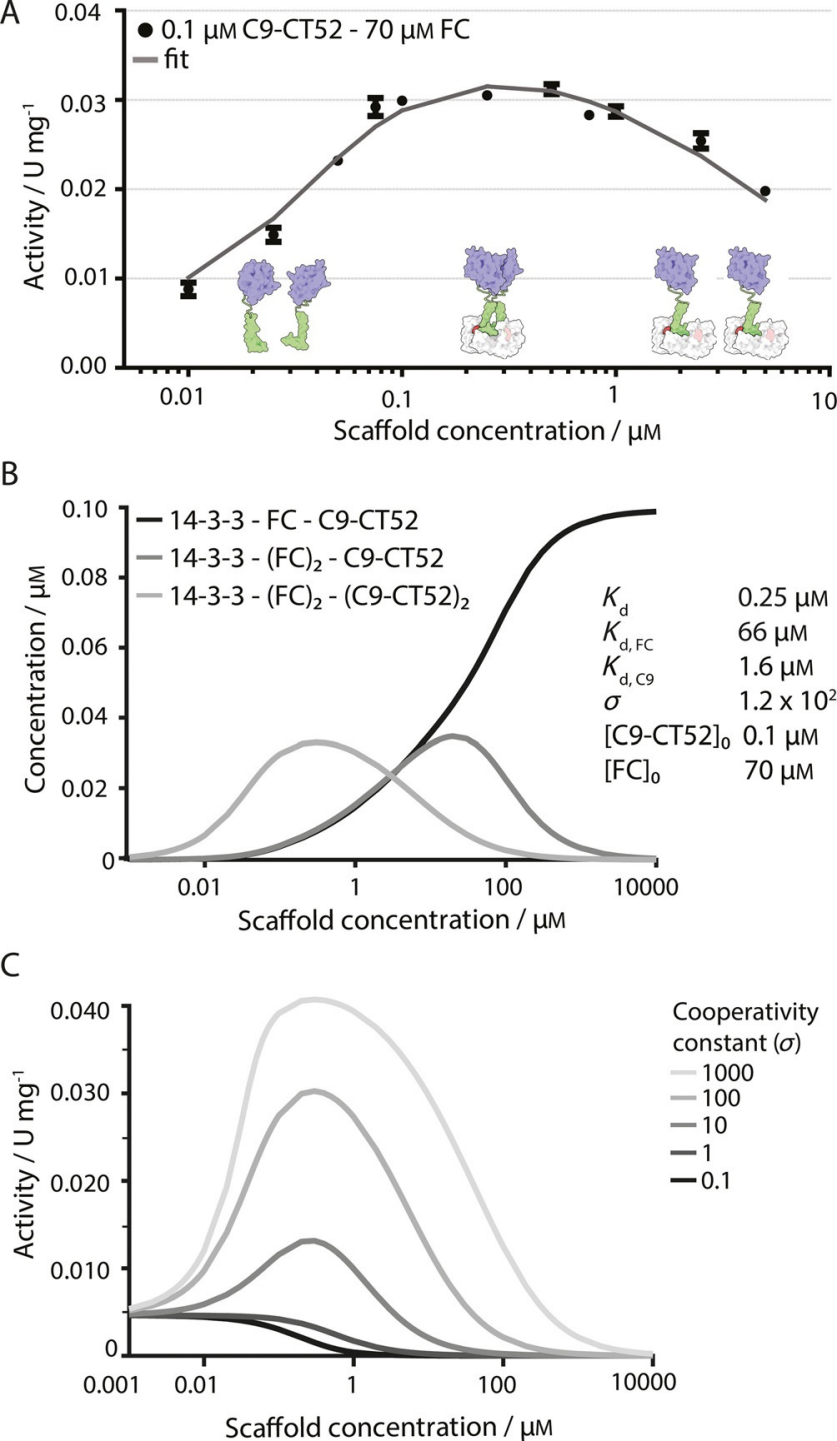
Cooperativity in 14‐3‐3 scaffolding of C9‐CT52. A) Activity assay with synthetic substrate Ac‐LEHD‐AFC (200 μm) at constant C9‐CT52 concentration (0.1 μm) and varying 14‐3‐3 concentrations (0.1–5 μm) in the presence of FC in excess (70 μm). Data points are experimentally determined at 37 °C in assay buffer (20 mm Na_2_HPO_4_, 150 mm NaCl, 1 mm EDTA, 2 mm TCEP, pH 7.0). Each error bar represents the standard deviation (*n*=3). The solid line represents the fit of the mathematical model with use of a *K*_d,FC_ of 66 μm, resulting in the determined parameters: binding affinity of C9‐CT52 for 14‐3‐3 (*K*_d_=0.25 μm), the cooperativity constant (*σ*=121.5), and dimerisation affinity for two C9‐CT52 units (*K*_d,C9_=1.64 μm). B) Overview of calculated steady‐state concentrations of assembled species plotted against initial 14‐3‐3 scaffold concentration for the stated parameters. C) C9‐CT52 activity (U mg^−1^) over various scaffold concentrations simulated at varying cooperativity constants (*σ*=0.1–1000).

Scaffold proteins should typically feature a biphasic effect, also known as combinatorial inhibition, characterised by an inverted dependence of enzyme activity at high scaffold concentrations.^27^, ^28^ This phenomenon is due to the fact that binding of two proteins to a scaffold results in the formation of the ternary complex at an optimal scaffold/protein stoichiometry, whereas suprastoichiometric scaffold protein levels promote the formation of enzymatically inactive binary complexes. The relationship between functional ternary complex and scaffold concentration thus depends on, amongst other factors, the cooperativity between the proteins when bound on the scaffold.^29^, ^30^ The larger the cooperativity for C9‐CT52 dimer formation on the scaffold, the lower the sensitivity to combinatorial inhibition. The C9‐CT52 enzymatic activity was therefore determined at varying 14‐3‐3 scaffold concentrations from 0.01 to 5 μm at a constant concentration of C9‐CT52 (0.1 μm) and with FC in excess (Figure 3 A). Maximum activity was achieved at a 14‐3‐3 scaffold concentration around 0.2 μm. Importantly, this high C9‐CT52 activity was more or less constant over the 0.08 to 1 μm concentration regime of the 14‐3‐3 scaffold. Only at scaffold concentrations much higher than the C9‐CT52 concentration was a clear decrease in enzyme activity observed. These results suggest strong cooperative binding of two C9‐CT52 units on the 14‐3‐3 scaffold.

To quantify the magnitude of this cooperativity and to allow further insight into the parameters that determine the scaffolding properties of our system, we developed a mathematical model that describes the assembly of C9‐CT52 on 14‐3‐3 under the influence of the small‐molecule compound FC and also takes non‐templated dimerisation of C9‐CT52 into account (Supporting Information). Nonlinear least‐squares optimisation was performed on multiple datasets to yield estimated values for *K*_d_, the binding strength of monovalent C9‐CT52 to 14‐3‐3 in the presence of FC, and *σ*, the cooperativity parameter describing the enhanced affinity of binding of the second C9‐CT52 monomer. The parameter estimation resulted in a *K*_d_ value of 0.25 μm, in accordance with values established before,^18^ and a *σ* value equal to 120, corroborating the strong positive cooperativity as already indicated by the broad plateau in enzyme activity (Figure 3 A). This strong cooperativity reflects the preference of C9 for formation of homodimers on the 14‐3‐3 platform, normally facilitated by appended protein domains,^21^, ^31^ which might be further enhanced by a weak intrinsic affinity between the appended CT52 elements.^15^

The mathematical model allows the calculation of the steady‐state concentrations of assembled 14‐3‐3–C9‐CT52 species for the determined parameters at various scaffold concentrations (Figure 3 B). The speciation plots reveal that formation of the active complex, consisting of 14‐3‐3 scaffold with two FC molecules and two C9‐CT52 monomer units (light grey line), is most abundant at scaffold concentrations in the range of the experimental conditions used, in line with the observed plateau between 0.08 and 1 μm 14‐3‐3 and with the scaffolding nature of 14‐3‐3 dimers.^32^ Only at 14‐3‐3 concentrations above 10 μm does combinatorial inhibition become prominent, resulting mainly in species consisting of the 14‐3‐3 scaffold bound to a single C9‐CT52 protein (Figure 3 B, dark grey and black line).

To elucidate the influence of the cooperativity on the activity plateau and combinatorial inhibition at high scaffold concentrations, we varied this critical parameter in our model (Figure 3 C). In the absence of scaffold, the typical C9 background activity can be seen.^19^, ^21^ The simulation reveals that higher *σ* values lead to overall higher enzymatic activity, because of a higher concentration of dimeric C9‐CT52. However, *σ* values above 100 have only minor additive importance. As well as an increase in the maximal activity, higher cooperativity also leads to broadening of the bell‐shaped curve. At *σ*=100 an activity plateau is observed over a protein concentration regime relevant for biochemical and cellular settings. In this concentration regime small variations in 14‐3‐3 scaffold concentration will thus only have a minor effect on activity, as can also be observed in Figure 3 A. Increasing the cooperativity further (*σ*=1000) leads to broadening of the bell‐shape profile towards higher, but biologically less relevant, 14‐3‐3 scaffold concentrations.

## Conclusion

In conclusion, we have delineated for the first time, in a bottom‐up approach and with a mathematical model based on quantitative in vitro data, the physical/chemical parameters of a robust and versatile scaffold protein system. This is also the first example of the concept of combinatorial inhibition under the control of a small‐molecule compound and for the 14‐3‐3 scaffold, thus harbouring great potential for implementing synthetic signalling systems based on this approach. The mathematical modelling provides insight into the parameters that determine the combinatorial inhibition of the 14‐3‐3 scaffold, revealing, amongst other things, strong cooperativity in C9‐CT52 activation. Overall, this leads to optimal caspase‐9 activity over a broad 14‐3‐3 concentration regime. The resulting descriptive mathematical model offers the potential for translation to other scaffold‐based systems.

## Supporting information

As a service to our authors and readers, this journal provides supporting information supplied by the authors. Such materials are peer reviewed and may be re‐organized for online delivery, but are not copy‐edited or typeset. Technical support issues arising from supporting information (other than missing files) should be addressed to the authors.

Supplementary
