# Supplementary material for: Small‐Molecule‐Induced and Cooperative Enzyme Assembly on a 14‐3‐3 Scaffold
Source: Chembiochem. 2016 Dec 27;18(3):331–5. doi: 10.1002/cbic.201600631 (PMC5299510; doi:10.1002/cbic.201600631)
Supplement: Supplementary file 1 — Supplementary [file CBIC-18-331-s001.pdf]

CHEMBIOCHEM

Supporting Information

Small-Molecule-Induced and Cooperative Enzyme Assembly on a 14-3-3 Scaffold

Anniek den Hamer, Lenne J. M. Lemmens, Minke A. D. Nijenhuis, Christian Ottmann, Maarten Merkx, Tom F. A. de Greef,* and Luc Brunsveld*[a]

cbic_201600631_sm_miscellaneous_information.pdf

Table of Contents

Protein expression and purification T14-3-3cΔC 2

Sequence T14-3-3cΔC 2

SDS-PAGE gel T14-3-3cΔC 2

Protein expression and purification C9-CT52 3

Sequence C9-CT52 3

SDS-PAGE gel C9-CT52 4

Activity assay synthetic substrate Ac-LEHD-AFC 5

Calibration curve AFC-standard 5

Protein expression and purification caspase-3-C158A 6

Sequence caspase-3-C158A 6

SDS-PAGE gel caspase-3-C158A 6

Activity assay natural substrate caspase-3-C158A 7

Kinetic model of scaffold-mediated enzyme activity under small-molecule control 8

Protein expression and purification T14-3-3cΔC

The 14-3-3 protein, encoded in a pOPINF plasmid[1], was expressed under sterile conditions in *E.coli BL21*-CodonPlus(DE3)-RIL (Novagen). Two liter cultures of LB medium were used in 5 L baffled conical flask and supplemented with ampicillin (100 µg/mL) and chloramphenicol (50 µg/mL) inoculated with 25 mL of overnight culture. The culture was incubated at 37 °C and 160 rpm until the optical density OD600 reached 0.6-0.8. Subsequently 0.5 mM isopropyl-β-D-thiogalactopyranoside (IPTG) was added to induce expression and the culture was incubated overnight at 18 °C. Cells were harvested by centrifugation (6,913 g, 10 min, 4 °C) in a Sorvall Evolution Centrifuge with a SLC-300 rotor (Thermo Scientific). The cell pellet was stored at -80° until purification, successively the pellet was resuspended in lysis buffer (10 mL per gram cell pellet, 50 mM Tris, 300 mM NaCl, 20 mM Imidazole, 2 mM TCEP, 5 mM $MgCl_2$, Benzonase® Nuclease (25 U per 10 mL buffer, Novagen) pH 8.0) and cells were lysed using a EmulsiFlexC3 High Pressure homogenizer (Avestin) at 15.000 psi for two rounds. Cell debris was removed by centrifugation (43,206 g, 45 min, 4 °C) in a Sorvall Evolution Centrifuge with a SA300 rotor. The supernatant was applied to a Ni-loaded column (His-Bind® Resin, Novagen) and washed with wash buffer (50 mM Tris, 300 mM NaCl, 20 mM Imidazole, 2 mM TCEP, pH 8.0) in presence and subsequently absence of 0.1% Triton-X-100. Protein was eluted from the column by elution buffer (50 mM Tris, 300 mM NaCl, 250 mM Imidazole, 2 mM TCEP, pH 8.0). Elution fractions were pooled and concentrated using Amicon Ultra Centrifugal Filters (MWCO 10 kDa, Millipore). Concentrated protein was bufferexchanged into assay buffer (20 mM Na_2HPO_4, 150 mM NaCl, 1 mM EDTA, 2 mM TCEP, pH 7.0) using a PD-10 desalting column (GE Healthcare). Concentration of the protein was determined using a Thermo Scientific ND-1000 spectrophotometer at 280 nm (ε = 27,390 M^{-1} cm^{-1} in water under reducing conditions).

Sequence T14-3-3cΔC

```
        10         20         30         40         50         60
MAHHHHHHSS GLEVLFQGMA VAPTAREENV YMAKLAEQAE RYEEMVEFME KVSNSLGSEE

        70         80         90        100        110        120
LTVEERNLLS VAYKNVIGAR RASWRIISSI EQKEESRGNE EHVNSIREYR SKIENELSKI

       130        140        150        160        170        180
CDGILKLLDA KLIPSAASGD SKVFYLKMKG DYHRYLAEFK TGAERKEAAE STLTAYKAAQ

       190        200        210        220        230        240
DIATTELAPT HPIRLGLALN FSVFYYEILN SPDRACNLAK QAFDEAIAEL DTLGEESYKD

       250
STLIMQLLRD NLTLWTSD
```

SDS-PAGE gel T14-3-3cΔC

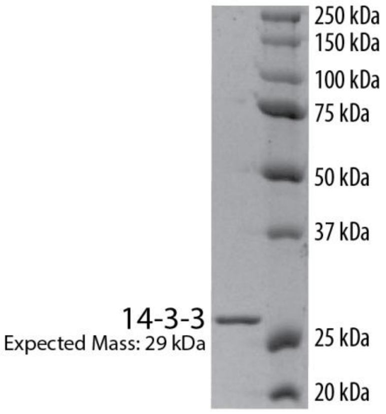

Figure S.1 – SDS-PAGE gel (4-20% Mini-PROTEAN® TGX^{TM} Precast Protein Gel – Bio-Rad) of purified T14-3-3cΔC with an expected mass of 29 kDa.

Protein expression and purification C9-CT52

The C9-CT52 protein and mutants, encoded in a pET28a plasmid, were expressed under sterile conditions in *E. coli* BL21 (DE3) (Novagen). Two liter cultures of LB medium were used in 5 L baffled conical flask and supplemented with kanamycin (30 µg/mL) inoculated with 25 mL of overnight culture. The culture was incubated at 37 °C and 160 rpm until the optical density OD_{600} reached 0.6-0.8. Subsequently 0.5 mM isopropyl-β-D-thiogalactopyranoside (IPTG) was added to induce expression and the culture was incubated overnight at 18 °C. Cells were harvested by centrifugation (6,913 g, 10 min, 4 °C) in a Sorvall Evolution Centrifuge with a SLC-300 rotor (Thermo Scientific). The cell pellet was stored at -80° until purification, successively the pellet was resuspended in lysis buffer (10 mL per gram cell pellet, PBS, 370 mM NaCl, 10% glycerol, 20 mM Imidazole, 0.1 mM TCEP, Benzonase® Nuclease (25 U per 10 mL buffer, Novagen), pH 7.4) and cells were lysed using a EmulsiFlexC3 High Pressure homogenizer (Avestin) at 15,000 psi for two rounds. Cell debris was removed by centrifugation (43,206 g, 45 min, 4 °C) in a Sorvall Evolution Centrifuge with a SA300 rotor. The supernatant was applied to a Ni-loaded column (His-Bind® Resin, Novagen) and washed with wash buffer (PBS, 370 mM NaCl, 10% glycerol, 20 mM Imidazole, 0.1 mM TCEP pH 7.4) in presence and subsequently absence of 0.1% Triton-X-100. Protein was eluted from the column by elution buffer (PBS, 370 mM NaCl, 10% glycerol, 250 mM Imidazole, 0.1 mM TCEP pH 7.4). Elution fractions were pooled and cleavage of the His_6-SUMO tag was performed in a dialysis bag (MWCO 3.5 kDa, Spectra Laboratories) in the presence of SUMO protease dtUD1 (purified according to reported protocol[2]) (1:500) and dialyzed against 4L of dialysis buffer(150 mM NaCl, 50 mM Tris, pH 7.8) stirring at 4 °C. The dialysis sample was further purified using a second Ni-column equilibrated with wash buffer and the flow-through containing the protein was collected. The protein was concentrated using Amicon Ultra Centrifugal Filters (MWCO 10 kDa, Millipore). Concentrated protein was bufferexchanged into assay buffer (20 mM Na_2HPO_4, 150 mM NaCl, 1 mM EDTA, 2 mM TCEP, pH 7.0) using a PD-10 desalting column (GE Healthcare). Concentration of the protein was determined using a Thermo Scientific ND-1000 spectrophotometer at 280 nm (ε = 25,440 M^{-1} cm^{-1} in water under reducing conditions).

Sequence C9-CT52

```
        10         20         30         40         50         60
MGSSHHHHHH SSGLVPRGSH MGGSDSEVNQ EAKPEVKPEV KPETHINLKV SDGSSEIFFK

        70         80         90        100        110        120
IKKTTPLRRL MEAFAKRQGK EMDSLRFLYD GIRIQADQAP EDLDMEDNDI IEAHREQIGG

       130        140        150        160        170        180
GALESLRGNA DLAYILSMEP CGHCLIINNV NFCRESGLRT RTGSNIDCEK LRRRFSSLHF

       190        200        210        220        230        240
MVEVKGDLTA KKMVLALLEL AQQDHGALDC CVVVILSHGC QASHLQFPGA VYGTDGCPVS

       250        260        270        280        290        300
VEKIVNIFNG TSCPSLGGKP KLFFIQACGG EQKDHGFEVA STSPEDESPG SNPEPDATPF

       310        320        330        340        350        360
QEGLRTFDQL DAISSLPTPS DIFVSYSTFP GFVSWRDPKS GSWYVETLDD IFEQWAHSED

       370        380        390        400        410        420
LQSLLLRVAN AVSVKGIYKQ MPGCFNFLRK KLFFKTSGGS GGSGGSGGSG GSGGSGGSGG

       430        440        450        460        470
SGGSGGSTNF NELNQLAEEA KRRAEIARQR ELHTLKGHVE SVVKLKGLDI ETIQQSYDI
```

*C268A and D478R were mutated using a QuikChange Lightning Multi-Site Directed Mutagenesis kit from Agilent using the following primers:

- C268A 5' GTTTTTCATCCAGGCGGCAGGCGGTGAACAAAAAG 3'
- D478R 5' ATCGAAACCATCCAACAATCGTATCGCATTTAACTCGAGCACCACCACC 3'

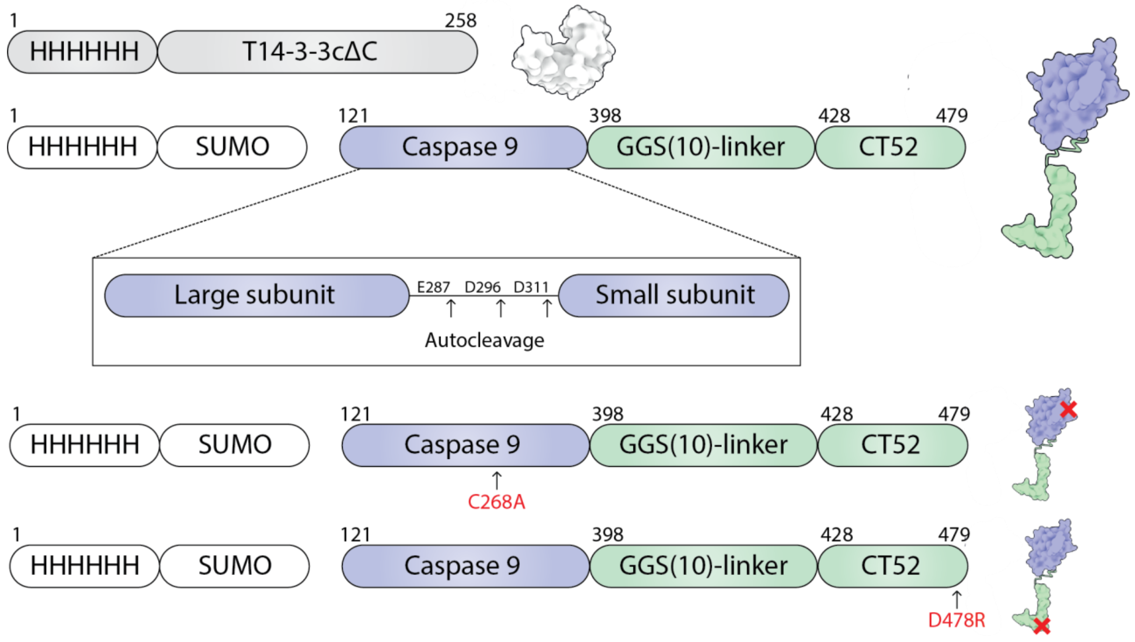

Figure S.2 – Schematic representation of used fusion proteins including auto-cleavage and mutation sites.

SDS-PAGE gel C9-CT52

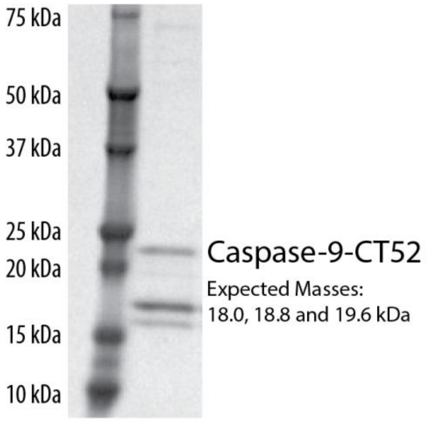

Figure S.3 – SDS-PAGE gel (4-20% Mini-PROTEAN® TGX^{TM} Precast Protein Gel – Bio-Rad) of purified C9-CT52, showing auto-cleavage in three parts, 18.0, 18.8 and 19.6 kDa.

Activity assay synthetic substrate Ac-LEHD-AFC

Activity assays with synthetic substrate Ac-LEHD-AFC (Enzo Life Sciences, dissolved as 10 mM stock in DMSO) were performed in activity assay buffer (20 mM Na_2HPO_4, 150 mM NaCl, 1 mM EDTA, 2 mM TCEP at pH 7.0). Assay components 0.02-10 µM 14-3-3 monomer, 0.1 µM C9-CT52 and 0.1 -1 µM FC (dissolved as 14.7 mM stock in EtOH, Enzo Life Sciences) were incubated for 30 minutes at room temperature to allow complex formation and subsequently substrate was added to a final volume of 100 µL. Measurements were done in a 96-well plate (Greiner F-bottom/chimney, black) at 37 °C, using an excitation wavelength of 400 nm (band with 20 nm) and an emission wavelength of 520 nm (band with 25 nm) using a Tecan Infinite F500 plate reader. Activity (U/mg) was calculated using the calibration curve below.

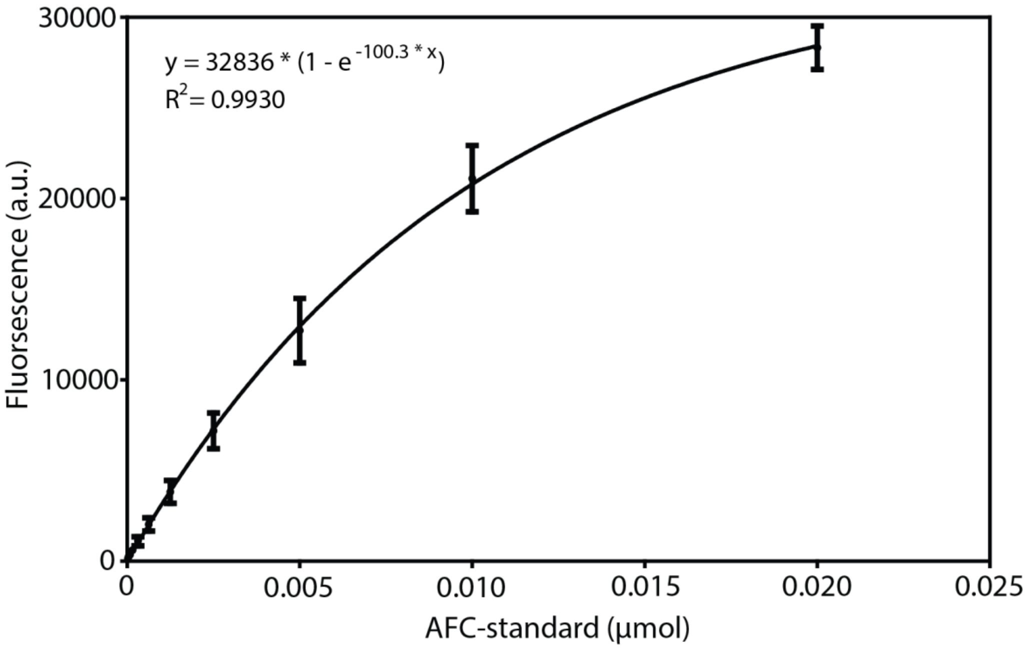

Calibration curve AFC-standard

Figure S.4 – Calibration curve of AFC standard whereby the total amount of cleaved AFC can be calculated by the formula below. Error bars represent the standard deviation of three independent measurements. The Fluorescence/min can then be converted to the catalytic rate (µmol/min; U) and subsequently be divided by the amount of caspase-9 used, to obtain the activity in U/mg.

$$\text{AFC}_{\text{cleaved}} = \frac{\ln(1 - \frac{Fluorescence}{32836})}{-100.3}$$

Protein expression and purification caspase-3-C158A

The caspase-3-C158A protein, encoded in a pET28a plasmid, was expressed under sterile conditions in *E. coli* BL21 (DE3) (Novagen). Two liter cultures of LB medium were used in 5 L baffled conical flask and supplemented with kanamycin (30 µg/mL) inoculated with 25 mL of overnight culture. The culture was incubated at 37 °C and 160 rpm until the optical density OD_{600} reached 0.6-0.8. Subsequently 0.5 mM isopropyl-β-D-thiogalactopyranoside (IPTG) was added to induce expression and the culture was incubated overnight at 18 °C. Cells were harvested by centrifugation (6,913 g, 10 min, 4 °C) in a Sorvall Evolution Centrifuge with a SLC-300 rotor (Thermo Scientific). The cell pellet was stored at -80° until purification, successively the pellet was resuspended in lysis buffer (10 mL per gram cell pellet, 20 mM Tris, 500 mM NaCl, 20 mM Imidazole, 2 mM TCEP, Benzonase® Nuclease (25 U per 10 mL buffer, Novagen), pH 7.4) and cells were lysed using a EmulsiFlexC3 High Pressure homogenizer (Avestin) at 15.000 psi for two rounds. Cell debris was removed by centrifugation (43,206 g, 45 min, 4 °C) in a Sorvall Evolution Centrifuge with a SA300 rotor. The supernatant was applied to a Ni-loaded column (His-Bind® Resin, Novagen) and washed with wash buffer (20 mM Tris, 500 mM NaCl, 20 mM Imidazole, 2 mM TCEP, pH 7.4) in presence and subsequently absence of 0.1% Triton-X-100. Protein was eluted from the column by elution buffer (20 mM Tris, 500 mM NaCl, 250 mM Imidazole, 2 mM TCEP, pH 7.4). Elution fractions were pooled and applied to a Strep-tactin column (IBA Life Sciences), the column was washed with wash buffer (100 mM Tris, 150 mM NaCl, 1 mM EDTA, pH 8.0) and subsequently the protein was eluted using elution buffer (100 mM Tris, 150 mM NaCl, 1 mM EDTA, 2.5 mM desthiobiotin, pH 8.0). Protein was buffer exchanged into assay buffer (20 mM Na_2HPO_4, 150 mM NaCl, 1 mM EDTA, 2 mM TCEP, pH 7.0) using a PD-10 desalting column (GE Healthcare). Concentration of the protein was determined using a Thermo Scientific ND-1000 spectrophotometer at 280 nm (ε = 31,400 M^{-1} cm^{-1} in water under reducing conditions).

Sequence caspase-3-C158A

```
        10         20         30         40         50         60
MGSSHHHHHH SSGLVPRGSH MASSGISLDN SYKMDYPEMG LCIIINNKNF HKSTGMTSRS

        70         80         90        100        110        120
GTDVDAANLR ETFRNLKYEV RNKNDLTREE IVELMRDVSK EDHSKRSSFV CVLLSHGEEG

       130        140        150        160        170        180
IIFGTNGPVD LKKITNFFRG DRCRSLTGKP KLFIIQAARG TELDCGIETD SGVDDDMACH

       190        200        210        220        230        240
KIPVEADFLY AYSTAPGYYS WRNSKDGSWF IQSLCAMLKQ YADKLEFMHI LTRVNRKVAT

       250        260        270        280
EFESFSFDAT FHAKKQIPCI VSMLTKELYF YHGGWSHPQF EK
```

* C158A was incorporated in the construct to obtain an inactive Caspase-3

SDS-PAGE gel caspase-3-C158A

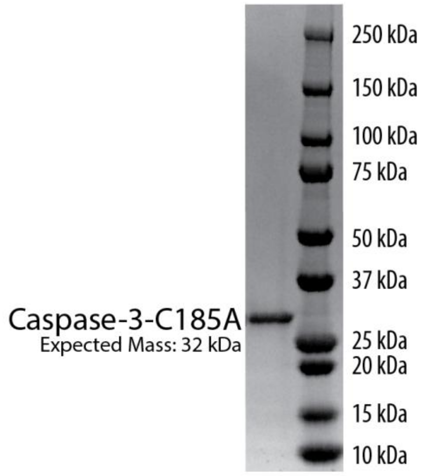

Figure S.5 - SDS-PAGE gel (4-20% Mini-PROTEAN® TGX™ Precast Protein Gel – Bio-Rad)of purified Caspase-3-C158A with an expected mass of 32 kDa.

Activity assay natural substrate caspase-3-C158A

Assay components (1 µM 14-3-3, 0.1 µM C9-CT52 and 1 µM FC) were incubated for 30 minutes at room temperature. Subsequently, 4 µM Caspase-3-C158A was added and incubated at 37°C in assay buffer (20 mM Na_2HPO_4, 150 mM NaCl, 1 mM EDTA, 2 mM TCEP, pH 7.0) at a final volume of 200 µL. Samples were taken at various time-points and the reaction was stopped by the addition of 2x SDS-loading buffer (125 mM Tris, 5% SDS, 20 % glycerol, 0.02% bromophenol blue at pH 6.8). Results were analysed on precast SDS-PAGE gels (4-20% Mini-PROTEAN® TGX^{TM} Precast Protein Gel – Bio-Rad) and protein was stained using Coomassie Brilliant Blue (R-250 Bio-Rad). Gel pictures were taken using the ImageQuant350 (GE Healthcare). Quantitation was done using Image Studio Lite v4.0.

Kinetic model of scaffold-mediated enzyme activity under small-molecule control

<u>Derivation and numerical solution</u>

Monovalent caspase-9 fusion proteins are recruited onto bivalent 14-3-3 scaffolds in the presence of the small molecule FC. In the kinetic model, the ternary complex consisting of two caspase-9 monomers bound to the bivalent 14-3-3/FC scaffold is assumed to be enzymatically active. In addition, caspase-9 monomers can homo-dimerize in a scaffold-independent fashion resulting in catalytically active dimers. Figure S7 shows a schematic representation of all the scaffold-mediated and scaffold independent equilibria involved as well as the catalytically active species.
Our kinetic model is based on a two-step procedure. The main assumption of the model is that equilibration of the monovalent C9-CT52, FC and 14-3-3 species is much faster than the catalytic conversion of the substrate. Therefore, in the first step the equilibrium concentration of all species is found by solving algebraic expressions for the equilibrium concentrations of each of the different species as a function of the parameters (the equilibrium constants and the total concentrations). In order to verify these assumptions, we have modeled the scaffold equilibration using realistic kinetic parameters which reveal that this assumption only leads to small errors (typically between 5-10%). We have incorporated the possibility for cooperative binding of the second monovalent caspase-9 fusion protein as many scaffold-mediated protein complexes display some level of cooperativity.[3] In contrast, binding of the small molecule FC to two binding sites on the 14-3-3 scaffold occurs in an independent fashion.
In the second step, we model the catalytic conversion of the substrate by recruited caspase dimers on the 14-3-3 scaffold as well as free caspase-9 homo-dimers by assuming that the rate of product formation can be described by a Michaelis-Menten approximation.
Let us define the following quantities:

C_{tot} = initial and total concentration of *C9-CT52* in mol·L^{-1}
F_{tot} = initial and total concentration of *FC* in mol·L^{-1}
B_{tot} = initial and total concentration of *14-3-3* scaffold in mol·L^{-1}
$K_{d,FC}$ = dissociation equilibrium constant for the binding of a single *FC* to the *14-3-3* scaffold in mol·L^{-1}
$K_{d,C9}$ = dissociation equilibrium constant for the dimerization of *C9-CT52* in mol·L^{-1}
K_d = dissociation equilibrium constant for the binding of a single C9-CT52 to a scaffold·*FC(·FC)* complex in mol·L^{-1}
σ = dimensionless cooperativity parameter for the binding of two identical C9-CT52 molecules to the same scaffold molecule
[C] = free concentration of *C9-CT52* in mol·L^{-1}
[F] = free concentration of *FC* in mol·L^{-1}
[B] = free concentration of *14-3-3* scaffold in mol·L^{-1}
[BF] = steady-state concentration of *scaffold·FC* complex in mol·L^{-1}
[BFF] = steady-state concentration of *scaffold·FC·FC* complex in mol·L^{-1}
[BFC] = steady-state concentration of *scaffold·FC·C9-CT52* complex in mol·L^{-1}
[BFFC] = steady-state concentration of *scaffold·FC·FC·C9-CT52* complex in mol·L^{-1}
[BFFCC] = steady-state concentration of enzymatically active ternary complex, *scaffold·FC·FC·C9-CT52·C9-CT52* in mol·L^{-1}
[CC] = steady-state concentration of enzymatically active *C9-CT52·C9-CT52* homo-dimer in mol·L^{-1}
K_M = Michaelis-Menten constant for enzymatically active *caspase-9* species in mol·L^{-1}
k_{cat} = catalytic rate constant for enzymatically active *caspase-9* species in s^{-1}
$[S]_0$ = initial concentration of substrate in mol·L^{-1}
[E] = total concentration of all available enzymatically active compounds (*scaffold·FC·FC·C9-CT52·C9-CT52* and *C9-CT52·C9-CT52*) in mol·L^{-1}

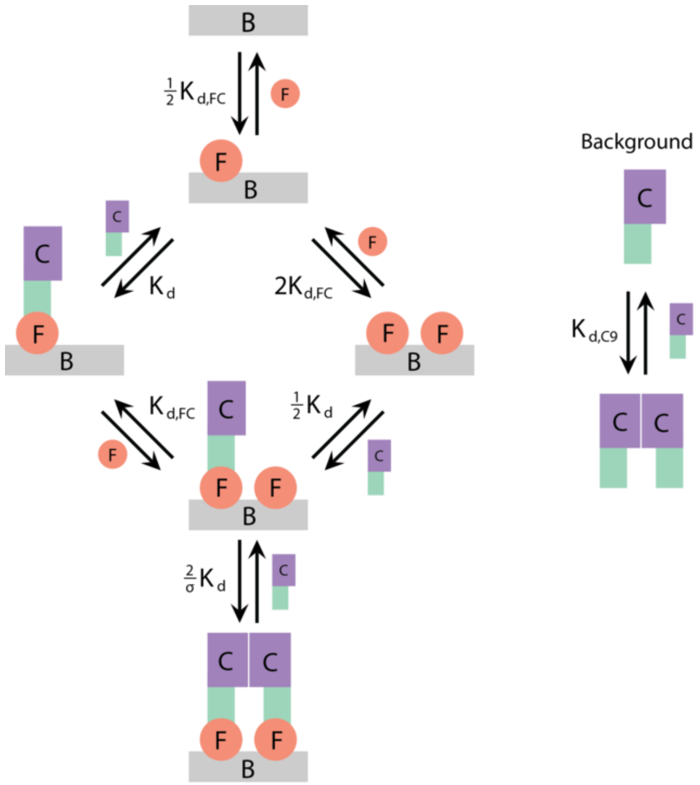

Figure S.7 shows a schematic representation of the various equilibria. When the scaffold-mediated recruitment of FC and monovalent caspase-9 has equilibrated, the initial concentration of *14-3-3* scaffold, B_{tot}, is partitioned into various complexes.

$$B_{tot} = [B] + [BF] + [BFF] + [BFC] + [BFFC] + [BFFCC] \quad \text{Eq. 1}$$

Next, we write down the mass-balances of the total concentration of *FC* and *C9-CT52* species partitioned between their respective free forms and various complexes (Eq. 2–3).

$$F_{tot} = [F] + [BF] + [BFC] + 2[BFF] + 2[BFFC] + 2[BFFCC] \quad \text{Eq. 2}$$

$$C_{tot} = [C] + [BFC] + [BFFC] + 2[BFFCC] + 2[CC] \quad \text{Eq. 3}$$

Now, we write down the equilibrium equations of the dissociation constant $K_{d,FC}$ and its related species, while taking into account the appropriate statistical factors. There are 3 possible reactions: the binding of *FC* to a free scaffold molecule, the binding of a second *FC* to a *BF* complex and the binding of *FC* to *BFC* complex (Eq. 4–6).

$$\frac{1}{2}K_{d,FC} = \frac{[F][B]}{[BF]} \quad \text{Eq. 4}$$

$$2K_{d,FC} = \frac{[BF][F]}{[BFF]} \quad \text{Eq. 5}$$

$$K_{d,FC} = \frac{[BFC][F]}{[BFFC]} \quad \text{Eq. 6}$$

Next, we derive expressions relating the *C9-CT52* containing species to the equilibrium constant K_d and the cooperativity parameter σ, while taking into account the appropriate statistical factors (Eq. 7–9). The homo-dimerization of *C9-CT52*, described by dissociation constant $K_{d,C9}$, is also considered (Eq. 10).

$$\frac{1}{2}K_d = \frac{[BFF][C]}{[BFFC]} \quad \text{Eq. 7}$$

$$\frac{2}{\sigma}K_d = \frac{[BFFC][C]}{[BFFCC]} \quad \text{Eq. 8}$$

$$K_d = \frac{[BF][C]}{[BFC]} \quad \text{Eq. 9}$$

$$K_{d,C9} = \frac{[C]^2}{[CC]} \quad \text{Eq. 10}$$

These equilibrium equations can be rewritten to obtain expressions for all complexes *BF, BFF, BFFC, BFFCC, BFC* and *CC* as a function of the dissociation constants relevant parameters and free concentrations of all species (Eq. 11–16.).

$$[BF] = \frac{2[F][B]}{K_{d,FC}} \quad \text{Eq. 11}$$

$$[BFF] = \frac{[F]^2[B]}{K_{d,FC}^2} \quad \text{Eq. 12}$$

$$[BFFC] = \frac{2[F]^2[B][C]}{K_{d,FC}^2 K_d} \quad \text{Eq. 13}$$

$$[BFFCC] = \frac{[F]^2[B][C]^2}{K_{d,FC}^2 K_d^2} \quad \text{Eq. 14}$$

$$[BFC] = \frac{2[F][B][C]}{K_{d,FC} K_d} \quad \text{Eq. 15}$$

$$[CC] = \frac{[C]^2}{K_{d,C9}} \quad \text{Eq. 16}$$

Finally, equations 11–16 are substituted in the mass-balance equations (eq. 1-3) to arrive at the following expressions:

$$B_{tot} = [B] + \frac{2[F][B]}{K_{d,FC}} + \frac{[F]^2[B]}{K_{d,FC}^2} + \frac{2[F][B][C]}{K_{d,FC}K_d} + \frac{2[F]^2[B][C]}{K_{d,FC}^2 K_d} + \frac{[F]^2[B][C]^2}{K_{d,FC}^2 K_d^2} \quad \text{Eq. 17}$$

$$F_{tot} = [F] + \frac{2[F][B]}{K_{d,FC}} + \frac{2[F][B][C]}{K_{d,FC}K_d} + \frac{2[F]^2[B]}{K_{d,FC}^2} + \frac{4[F]^2[B][C]}{K_{d,FC}^2 K_d} + \frac{2[F]^2[B][C]^2}{K_{d,FC}^2 K_d^2} \quad \text{Eq. 18}$$

$$C_{tot} = [C] + \frac{2[F][B][C]}{K_{d,FC}K_d} + \frac{2[F]^2[B][C]}{K_{d,FC}^2 K_d} + \frac{2[F]^2[B][C]^2}{K_{d,FC}^2 K_d^2} + \frac{2[C]^2}{K_{d,C9}} \quad \text{Eq. 19}$$

Custom-written Matlab scripts are used to solve the coupled non-linear equations 17-19 for the free concentrations *B*, *F* and *C*. These can subsequently be used to calculate the steady-state concentrations of all other species (eq11-16), most importantly the enzymatically active complexes *BFFCC* and *CC*. The total amount of enzymatically active compounds in steady-state, *E* and initial substrate concentration S_0 are used to find the initial reaction rate v_0 as given by the Michaelis-Menten equation (Eq. 20). Furthermore, a composite parameter was introduced, which represents all parameters that influence only the height of the activity curve (K_M, k_{cat} and S_0).

$$v_0 = \frac{k_{cat} \cdot [E] \cdot [S]_0}{K_M| + [S]_0} \quad \text{Eq. 20}$$

The initial reaction rate is converted into enzymatic activity measured in U/mg. For this, a conversion factor is used that contains the total *C9-CT52* concentration C_{tot}, the volume *V* of the experimental reaction mixture and the molecular weight *Mw* of *C9-CT52* (Eq. 21).

$$activity = v_0 \cdot \frac{6}{C_{tot} \cdot V \cdot Mw} \quad \text{Eq. 21}$$

<u>Data analysis and parameter estimation</u>

We performed non-linear least square analysis on the activity assay data as reported in Figure 3.A by comparing the activity data to the computed activity. Previous work has reported accurate values for $K_{d,FC}$ (66 μM[4]) and as such this constant was assumed constant during the non-linear least square optimization. To increase the accuracy of the estimated parameters, we used three datasets in the optimization obtained by scaffold titrations using different concentrations of FC (Figure 3.A and Figure S.8). During the fitting, a composite parameter was introduced, which incorporates all parameters that only influence the height of the activity curve (K_M, k_{cat} and S_0). S_0 is set at 200 μM according to the experimental parameters and the determined K_M (650 μM) and k_{cat} (0.2 s^{-1}) are both in accordance with literature.[5–7]

To compensate for length differences between the datasets, residuals were multiplied by a relative weight factor. Non-linear least square minimization of the data was performed using the Matlab function *lsqnonlin*, a subspace trust region method based on the interior-reflective Newton method. In order to prevent entrapment in a local minimum, 30 different starting values of K_d, σ, $K_{d,C9}$ and $\frac{k_{cat} \cdot [S]_0}{K_M + [S]_0}$ were defined, and the best fit (defined as the fit with the lowest squared 2-norm of the residuals) is taken as the final solution for the optimized values. The different initial parameter sets are defined using a *latin hypercube sampling* method (Matlab function *lhsdesign*). The standard deviation of the parameters were

calculated using the Fischer information matrix.[8] The optimized values of K_d, σ, $K_{d,C9}$ and $\frac{k_{cat} \cdot [S]_0}{K_M + [S]_0}$ and their standard errors are reported in Table S.1.

Table S.1 Optimized values and their standard deviation

K_d	$0.249 \pm 0.065 \times 10^{-6}$ M
σ	122 ± 69
$K_{d,C9}$	$1.64 \pm 1.49 \times 10^{-6}$ M
$\frac{k_{cat} \cdot [S]_0}{K_M + [S]_0}$	0.0060 ± 0.0010 s^{-1}

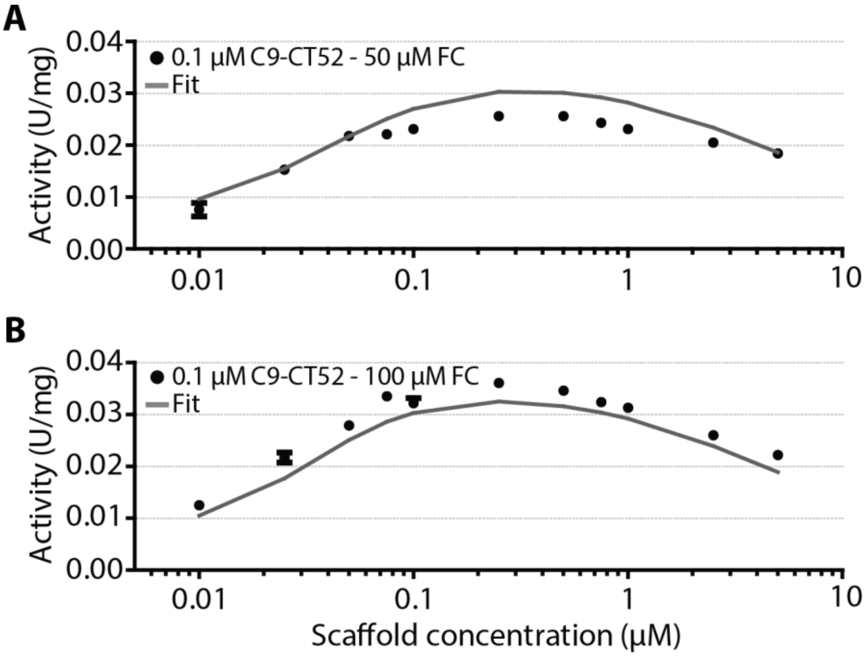

Figure S.8 Additional datasets for the non-linear least square optimization – according to Figure 3.A Activity Assay with synthetic substrate Ac-LEHD-AFC (200 µM) at constant C9-CT52 (0.1 µM) and varying 14-3-3 concentrations (0.1 – 5 µM) and excess of FC a) 50 µM and b) 100 µM. Data points are experimentally determined at 37 °C in assay buffer (20 mM Na_2HPO_4, 150 mM NaCl, 1 mM EDTA, 2 mM TCEP, pH 7.0). Error bars represent the standard deviation based on three independent measurements. The solid line represents the fit of the mathematical model.

Supporting References

[1] N. S. Berrow, D. Alderton, S. Sainsbury, J. Nettleship, R. Assenberg, N. Rahman, D. I. Stuart, R. J. Owens, *Nucleic Acids Res.* **2007**, *35*, DOI 10.1093/nar/gkm047.

[2] S. D. Weeks, M. Drinker, P. J. Loll, *Protein Expr. Purif.* **2007**, *53*, 40–50.

[3] J. Yang, W. S. Hlavacek, *Math. Biosci.* **2011**, *232*, 164–173.

[4] M. Würtele, C. Jelich-Ottmann, A. Wittinghofer, C. Oecking, *EMBO J.* **2003**, *22*, 987–94.

[5] Q. Yin, H. H. Park, J. Y. Chung, S. C. Lin, Y. C. Lo, L. S. da Graca, X. Jiang, H. Wu, *Mol. Cell* **2006**, *22*, 259–268.

[6] C. Pop, J. Timmer, S. Sperandio, G. S. Salvesen, *Mol. Cell* **2006**, *22*, 269–275.

[7] M. Garcia-Calvo, E. P. Peterson, D. M. Rasper, J. P. Vaillancourt, R. Zamboni, D. W. Nicholson, N. A. Thornberry, *Cell Death Differ.* **1999**, *6*, 362–9.

[8] W. H. Press, S. A. Teukolsky, W. T. Vetterling, B. P. Flannery, *Numerical Recipes - The Art of Scientific Computing*, Cambridge University Press, **2007**.
